# Magnetic Properties and THz Emission from Co/CoO/Pt and Ni/NiO/Pt Trilayers

**DOI:** 10.3390/nano14020215

**Published:** 2024-01-19

**Authors:** Nikolaos Kanistras, Laura Scheuer, Dimitrios I. Anyfantis, Alexandros Barnasas, Garik Torosyan, René Beigang, Ovidiu Crisan, Panagiotis Poulopoulos, Evangelos Th. Papaioannou

**Affiliations:** 1 Institute of Physics, Martin Luther University Halle-Wittenberg, Von-Danckelmann Platz 3, 06120 Halle, Germany; nikolaos.kanistras@physik.uni-halle.de; 2 Fachbereich Physik and Landesforschungszentrum OPTIMAS, Rheinland-Pfälzische Technische Universität Kaiserslautern-Landau, 67663 Kaiserslautern, Germany; scheuer@physik.uni-kl.de (L.S.); rene.beigang@icloud.com (R.B.); 3 Department of Materials Science, School of Natural Sciences, University of Patras, 26504 Patras, Greece; up1057157@upatras.gr (D.I.A.); mparnalex@gmail.com (A.B.); poulop@upatras.gr (P.P.); 4 Photonik Center Kaiserslautern, 67663 Kaiserslautern, Germany; garik.torosyan@pzkl.de; 5 National Institute of Materials Physics, Atomistilor 405A, 077125 Magurele, Romania; ocrisan@infim.ro; 6 Department of Physics, Aristotle University of Thessaloniki, 54124 Thessaloniki, Greece

**Keywords:** nanomagnetism, THz spintronics, antiferromagnetic films, exchange bias, transition metal oxides

## Abstract

THz radiation emitted by ferromagnetic/non-magnetic bilayers is a new emergent field in ultra-fast spin physics phenomena with a lot of potential for technological applications in the terahertz (THz) region of the electromagnetic spectrum. The role of antiferromagnetic layers in the THz emission process is being heavily investigated at the moment. In this work, we fabricate trilayers in the form of Co/CoO/Pt and Ni/NiO/Pt with the aim of studying the magnetic properties and probing the role of very thin antiferromagnetic interlayers like NiO and CoO in transporting ultrafast spin current. First, we reveal the static magnetic properties of the samples by using temperature-dependent Squid magnetometry and then we quantify the dynamic properties with the help of ferromagnetic resonance spectroscopy. We show magnetization reversal that has large exchange bias values and we extract enhanced damping values for the trilayers. THz time-domain spectroscopy examines the influence of the antiferromagnetic interlayer in the THz emission, showing that the NiO interlayer in particular is able to transport spin current.

## 1. Introduction

Spintronic THz materials are a new class of THz radiation sources [1,2]. They are based on ultrafast spin transport phenomena that are induced when a heterostructure composed of a ferromagnetic (FM) and non-magnetic layer (NM) is illuminated by femtosecond (fs) laser pulses. The latter excites a spin current in the FM layer which is transferred to the NM layer. The inverse spin Hall effect (ISHE) then converts the spin current to an ultrafast charge current that is able to emit THz radiation [1,2]. The so-called spintronic THz emitters (STEs) hold promise for the next generation of THz technologies, since they are able to provide high field strengths [3,4,5] and spatiotemporal modulation of the THz beam [6,7,8,9] and a very wide spectrum reaching up to 30 T Hz [1,10,11].

Many materials have been examined as potential STEs [12,13,14,15,16,17,18,19,20,21,22] and the knowledge gained so far shows that the most efficient emitters are magnetic/non magnetic bilayers as Fe/Pt, Co/Pt and trilayers like W/CoFeB/Pt.

Recent experiments have also tried to probe the effect when an interlayer is introduced at the FM/NM interface [23,24]. In these experiments, the spin current passes through additional interfaces between the magnetic material and the NM material, which in most of the cases is a Pt layer. The interlayer affects the spin current distribution through the introduction of different scattering events. The role of the interlayer is then reflected in the modified THz emission. Interlayers like MgO [25,26], Cu [23,27], Al [23], FePt [24], CoPt [28] alloys have been studied, showing strong modification of the spin-current transmission and the efficiency of the radiation, since the spin current has to pass through two interfaces instead of one.

In this work, we follow a different strategy concerning interlayers: at the FM/NM interface, we introduce an antiferromagnetic layer like that of NiO and CoO. Antiferromagnetic materials are a very interesting class of materials for THz spectroscopy, since they have magnon resonance frequencies in the THz region [29]. In the last few decades, the use of antiferromagnetic layers in spintronic structures has become the focus of research in the magnetic community. Initially, they were used to pin the magnetization in giant magnetoresistance sensors. Nowadays, the ultrafast dynamics of the antiferromagnets and their use in spintronic multilayers hold promise for new, faster devices that operate in the THz frequency range [30,31,32,33,34]. The main advantage of AFM layers is that they are robust against external magnetic fields, while the inherent spin oscillation frequency (antiferromagnetic resonance) extends into the terahertz regime; in other words, they have much faster spin dynamics than the typical GHz excitations of the ferromagnets. Furthermore, the ultrafast manipulation of the antiferromagnetic order parameter has the potential to control the ultrafast magnetization of an exchange-biased adjacent ferromagnetic layer. NiO and CoO AFM layers have attracted the interest of many studies. In particular, single crystals and polycrystalline NiO have been investigated using optical laser pulses and terahertz pulses. Indicative studies on the THz spin dynamics in antiferromagnetic NiO have been performed using the magnetic component of intense terahertz transients, which enabled the probing of the spin degree of freedom [35], or using continuous-wave (cw) frequency domain THz spectroscopy on monocrystalline and polycrystalline NiO pellets in which the resonant frequency of ≈1 THz was found, and the dependence of damping of the antiferromagnetic resonance for the different crystal qualities was revealed [36]. Coherent spin oscillations at 1.07 THz and 140 GHz were measured using a time-resolved pump-probe magnetooptical setup [37], while the optical pulses excitation with different polarization states led to excitation of AFM magnons in NiO(110) due to inverse Faraday effect [38]. In our approach, we do not aim to resonantly excite the AFM magnons in the AFM layers but to use AFM layers as a functional layer for the spin current transmission between FM/NM junctions. This direction has been probed in the literature for a variety of AFM layers, especially for the NiO case. In particular, spin pumping-inverse spin Hall effect experiments at GHz excitation frequencies of Y_3_Fe_5_5O_12_(YIG)/NiO/Pt suggested that the NiO thin layers enhanced the spin current driven into the Pt layer due to AFM magnons or AFM fluctuations [39]. Furthermore, epitaxial NiO (001) layers were fabricated in MgO/Pt/NiO/FeNi/SiO_2_ multilayers and spin-transfer ferromagnetic resonance (ST-FMR) experiments were performed which showed a highly efficient angular momentum transfer through the epitaxial NiO, a result that was attributed to the well-defined orientation of the antiferromagnetic moments and the spin quantization axis of the injected spin current from the Pt layer [40]. ST-FMR measurements were also used to quantify the magnon current in NiO in multilayers of Bi_2_Se_3_/NiO/Py, and it was shown that the magnon current in NiO is able to exert a magnon torque that is sufficient to control the magnetization of the magnetic Py layer [41]. Spin-pumping inverse spin Hall effect measurements have also been performed for the case of CoO in Y_3_Fe_5_5O_12_(YIG)/CoO/Pt [42], in which an enhancement of the inverse spin Hall voltage was recorded near the transition temperature from the AFM to the paramagnetic phase. The effect was attributed to spin fluctuations near the magnetic phase transition.

Here, we study trilayers in the form of Co/CoO/Pt and Ni/NiO/Pt with very thin CoO and NiO of 1.4 n m thickness. We examine the influence of the antiferromagnetic oxide layer in the magnetization dynamics and the magnetization reversal. Unlike in the aforementioned studies in the GHz excitation scheme, in our experiment, we excite the system with fs-laser optical pulses of 800 n m . We probe the possible pumping of the spin current from the FM layer generated by the optical pulses into the AFM and then into Pt by using THz time-domain spectroscopy (THz-TDS). Similarly to our work, CoO layers were stimulated by fs-laser pulses in FeCoB/CoO/Pt structures and the THz emission was detected due to transmission of spin current through the CoO on a picosecond timescale [43]. However in our work, in contrast to Sasaki et al. [43], the CoO and the NiO are not epitaxially grown on buffer layers. We test the concept of whether very thin polycrystalline CoO and NiO layers can still function in such spintronic THz emitters structures. We reveal that even antiferromagnetic very thin and polycrystalline layers can still operate as spin current transmitters, a fact that can be very advantageous for technological applications.

## 2. Materials and Methods

Co/CoO/Pt and Ni/NiO/Pt multilayers were fabricated using a high-vacuum radio-frequency (RF) magnetron sputtering system with a base pressure of $3\times 10^{-7}$ m bar . Pure argon 6N gas was used for sputtering. The Ar pressure was kept constant for both metals at $3\times 10^{-3}$ m bar . The Co and Pt deposition rates and RF power were 0.75 (30 W ) and 1.5 Ås^−1^ (20 W ), respectively. The film thickness was determined using a quartz balance system (Inficon XTM/2, Kurt J. Lesker Company, Jefferson Hills, PA, USA). MgO (100) and Si (001) and ${\mathrm{Al}}_{2}O_{3}$ polished on both sides were used as substrates. The ferromagnetic Co and Ni layer were directly deposited on each substrate, followed by the deposition of CoO and NiO oxides. After the deposition of the metallic layer, atmospheric air was allowed to flow into the system through a fine valve at a pressure of 2–3 $\times 10^{-3}$ mbar. This led to the formation of a thin passive-oxide layer on the surface of the metal. More information about the growth process can be found in reference [44]. The thickness of the FM layers were kept at 10 n m , where the thickness for both oxides was 1.4 n m . A Pt overlayer of 3 n m thickness was finally deposited. In short, the grown structures had thicknesses of ${\mathrm{Co}}_{10nm}/{\mathrm{CoO}}_{1.4nm}/{\mathrm{Pt}}_{3nm}$ and ${\mathrm{Ni}}_{10nm}/{\mathrm{NiO}}_{1.4nm}/{\mathrm{Pt}}_{3nm}$ .

The magnetic properties of the films were measured with the help of a Superconducting Quantum Interference Device magnetometer (SQUID-VSM, Quantum Design, Darmstadt, Germany) by using applied magnetic fields of up to 30 k Oe in a temperature range of 4–300 K .

For the spin dynamics study, we performed ferromagnetic resonance (FMR) measurements using the so-called field modulation FMR technique [45]. The ferromagnetic resonance was probed by a microwave magnetic field $H_{rf}$ induced by a coplanar waveguide, which was fed by a continuous-wave signal at constant frequency f in the range of 5–19 G Hz . A tunable static external magnetic field H was applied and modulated with 0.2–0.3 m T amplitude at a frequency of 197 Hz for a better signal-to-noise ratio. The RF transmission was measured using a diode and a lock-in technique was used to improve the signal-to-noise ratio. The sample was placed on top of the waveguide and the Pt layer was facing the antenna with a thin insulating layer between them.

The THz experiments were performed using a standard THz-TDS system, in which the trilayers were used as THz emitters [46]. The fsT i:Sa laser produces optical pulses of 22 f s length at a wavelength of 800 n m with a repetition rate of 75 M Hz and a typical average output power of 500 m W . The probe beam is used to excite a photoconductive antenna (PCA) that acts as a THz detector. The spintronic emitter is magnetized by a constant external magnetic field of maximum value of 20 m T .

## 3. Results

### 3.1. Magnetization Reversal

**Figure 1:**
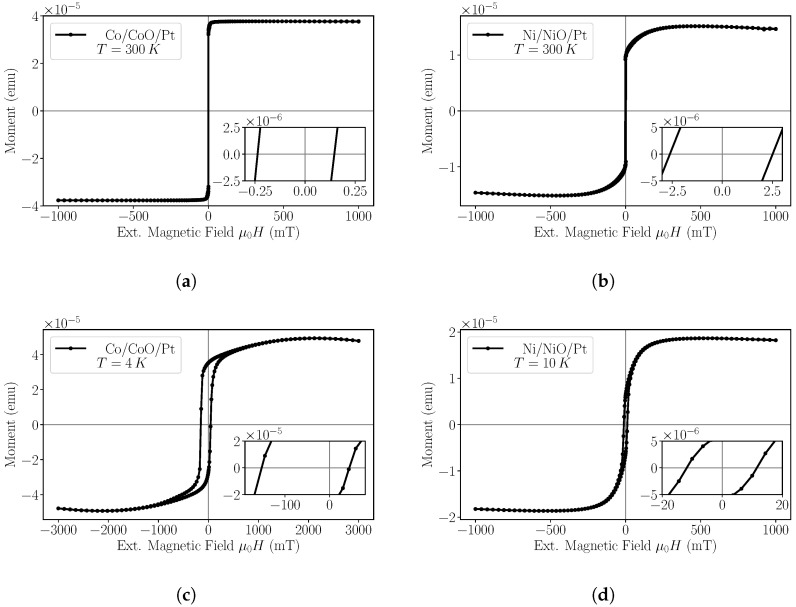
In-plane hysteresis curves obtained using Squid magnetometry. (**a**) CoCoOPt curves measured at T=300 K and (**b**) at T=4 K . (**c**) NiNiOPt hysteresis curve obtained at T=300 K and (**d**) at T=10 K .

To record the hysteresis loops with the SQUID-VSM magnetometer, we begin by setting the temperature and the maximum external magnetic field. We start by measuring from the maximum field value and the loop runs from the positive maximum saturation field to the negative maximum field and back to the positive maximum field. In order to calculate the exchange bias, we also record the loop for a second time by starting at the negative direction of the magnetic field. We estimate the exchange bias by adding the field values for zero magnetization (coercivity) $H_{C(+B)}$ + $H_{C(-B)}$ , where $H_{C(+B)}$ is the coercivity value for the positive magnetic field direction +B and $H_{C(-B)}$ is the coercivity value for the negative field direction. We have measured in-plane magnetization reversal in the temperature range from 4 K to 300 K . Hysteresis loops are indicatively shown in Figure 1 for both samples at 4 K , 10 K and 300 K .

Both Co- and Ni-based structures show a soft magnetic behaviour at room temperature reaching saturation at small external magnetic fields. The Co-based trilayer has an almost square loop at room temperature, while the Ni-based saturation is reached more gradually, which is indicative of a magnetic domain wall nucleation procedure.

At lower temperatures, the behaviour changes dramatically. The Co-based sample exhibits a large exchange bias (EB) effect [47], reaching field values of $H_{\mathrm{EB}}=110\;mT$ at 4 K . The exchange bias starts to appear at temperatures below 150 K . Furthermore, there is a large increase in the saturation field, which almost reaches the value of 2 T at a temperature of 4 K .

The Ni/NiO/Pt sample exhibits much smaller exchange bias values that are also noticeable below 150 K and they reach maximum values in the range of 0.4 m T at low temperatures. The S-shape loop is present in all temperatures, while a small hysteresis appears for the lowest temperatures. The saturation field exhibits small variation with temperature.

**Figure 2:**
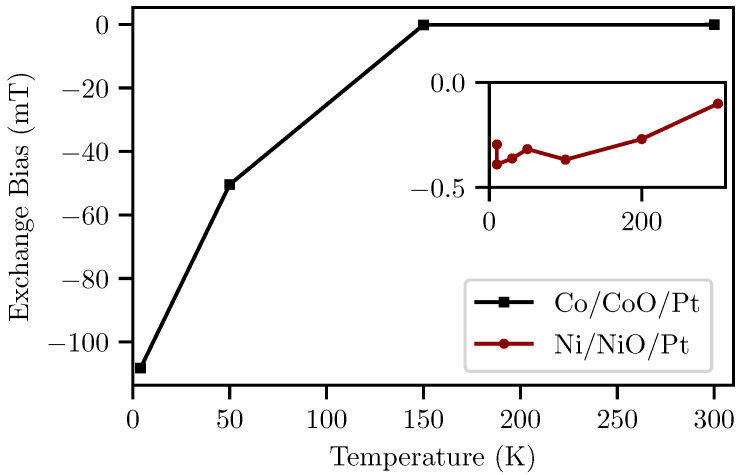
Exchange bias values for Co/CoO/Pt. The values have been calculated as the sum of coercivity values for the positive and the negative field direction, $H_{C(+B)}$ + $H_{C(-B)}$ . Below 150 K , the antiferromagnetic phase of CoO drives the exchange bias to very large values. (Inset) The inset shows the exchange bias values for Ni/NiO/Pt that obtain significant smaller values.

The evolution with the temperature of the EB values is shown in Figure 2. The CoO antiferromagnetic oxide seems to couple strongly with the FM layer below 150 K . The strong coupling is attributed to the fact that the CoO layer becomes an active antiferromagnet below 150 K . The onset of the appearance of EB is the so-called blocking temperature T_B_ [48]. The exchange bias further increases for lower temperatures. This is due to a stronger coupling between the FM and AFM layer. Furthermore, it is known that T_B_ is generally lower than the Néel temperature [49]. However, even at T > T_B_ the FM/AFM layers can be still coupled in cases in which the AFM order is only partially established due to the presence of different sizes of AFM grains [49]. Similarly, there is an increase in the EB at lower temperatures for the NiO case but the values are smaller compared to the CoO counterpart.

Bulk CoO has a Néel temperature close to room temperature, $T_{N-\mathrm{CoO}}=293\;K$ [50] while bulk NiO Néel temperature is $T_{N-\mathrm{NiO}}=525\;K$ [51]. However, finite size effects are able to reduce the Néel temperature [52]. In our case, the CoO and NiO layers are only 1.4 n m thickness. Such a low thickness can reduce the magnetic phase transition temperature below room temperature [52].

For both samples, the value of exchange bias at room temperature is almost zero within the experimental error, so we can claim that the FMR and THz experiments in the following sections at room temperature are performed in the non-strong coupling regime between FM-AFM layers. Despite the absence of EB at room temperature, the magnetic oscillations of the FM layer can still magnetically couple the neighboring NiO, CoO interfaces.

### 3.2. Ferromagnetic Resonance Spectroscopy, FMR

**Figure 3:**
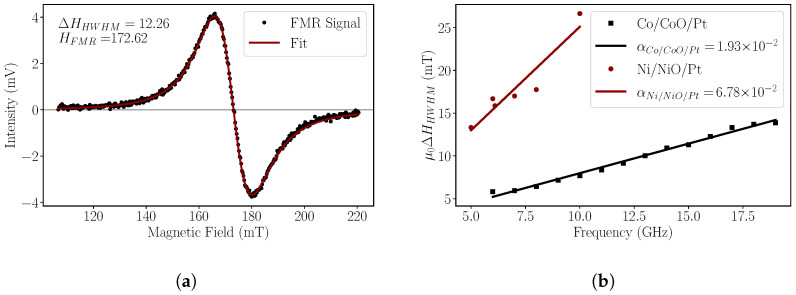
Results of FMR Spectroscopy. (**a**) Example of a differential FMR resonance curve recorded for the Co/CoO/Pt sample at constant frequency of 16 GHz. (**b**) Calculation of the damping parameter α according to Equation (2). For the Co/CoO/Pt equal to $\alpha =1.93\times 10^{-2}$ and for the Ni/NiO/Pt equal to $\alpha =6.78\times 10^{-2}$ .

Ferromagnetic resonance spectra were recorded for both systems at room temperature. Figure 3a shows a typical FMR absorption spectrum for a ${\mathrm{Co}}_{10nm}/{\mathrm{CoO}}_{1.4nm}/{\mathrm{Pt}}_{3nm}$ measured at a frequency of 16 G Hz . The field-modulation FMR technique yields differential absorption curves, as they are the first derivative of the susceptibility curve [45]. The red line is a result of the fitting procedure of the data using a differential Lorentzian fitting function according to (1) [53]:

(1) $$-A\frac{2\Delta H_{HWHM}\left(H-H_{FMR}\right)}{{\left(\Delta H_{HWHM}^{2}+{\left(H-H_{FMR}\right)}^{2}\right)}^{2}}+B\frac{\Delta H_{HWHM}^{2}-{\left(H-H_{FMR}\right)}^{2}}{{\left(\Delta H_{HWHM}^{2}+{\left(H-H_{FMR}\right)}^{2}\right)}^{2}}$$

where *H* is the applied external static magnetic field, $H_{FMR}$ is the resonance field, $\Delta H_{HWHM}$ is the linewidth at half width and at half maximum, and A and B are the symmetric and anti-symmetric coefficients. From the function (1), we are able to extract the resonance field, $H_{FMR}$ and the linewidth $\Delta H_{HWHM}$ (half width at half maximum). By further evaluating the data, we can quantify the Gilbert damping parameter α via the dependence of the linewidth, $\Delta H_{HWHM}$ , on the resonance frequency, $f_{res}$ , as shown in Figure 3b. The red line is a linear fit to Equation (2) [54]:

(2) $$\mu_{0}\Delta H_{HWHM}=\mu_{0}\Delta H_{0_{HWHM}}+\frac{2\pi \alpha f_{res}}{\gamma }\;$$

Here, $\Delta H_{0}$ is the inhomogeneous broadening and is related to the film quality, α the damping parameter to be estimated, γ the gyromagnetic ratio, $\mu_{0}$ the permeability of free space, $\Delta H_{HWHM}$ and $f_{res}$ are experimentally determined through many curves similar to those shown in Figure 3a.

The damping values appear higher than the reported values of single Co and Ni layers that are in the range of 1 × 10^−2^ [55,56].

Usually, the presence of a non-magnetic layer like Pt directly on top of a ferromagnetic film can increase the damping due to the spin-pumping effect, where the spin current produced during the FMR process is transferred to the adjacent non-magnetic layer. In our case, the generated spin current must pass through CoO and NiO very thin oxide layers. The larger values compared to single Co and Ni films hint to spin-current dissipation due to the overlayers. The magnetic measurements point out that the CoO couples strongly with the Co layer far below the room temperature at which the FMR measurements take place. Similarly, the Ni-NiO coupling is very weak and almost zero at room temperature. In short, Co/CoO/Pt and Ni/NiO/Pt exhibit significant damping values that can indicate dissipation of spin current to the adjacent layers during the FMR process.

### 3.3. THz Spectroscopy

**Figure 4:**
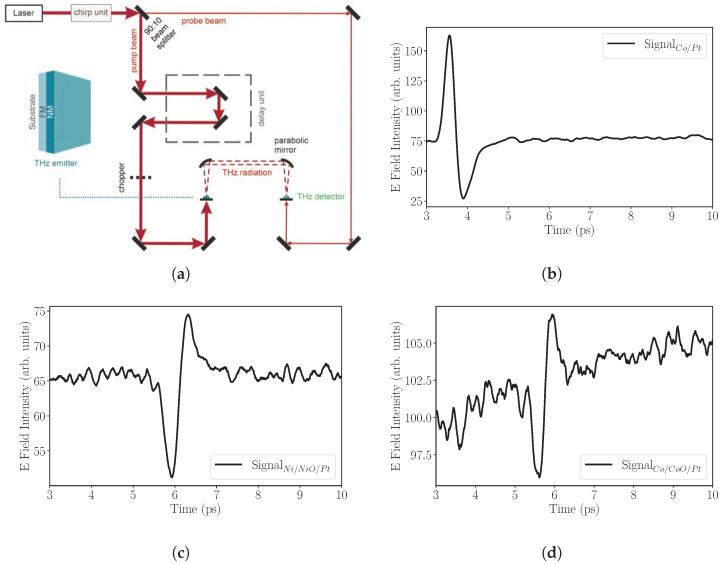
(**a**) THz-TDS system that was used in the measurements, (**b**) typical THz signal from a reference ${\mathrm{Co}}_{10nm}/{\mathrm{Pt}}_{3nm}$ sample grown on ${\mathrm{Al}}_{2}O_{3}$ substrate. (**c**,**d**) THz spectra from MgO/Co/CoO/Pt and MgO/Ni/NiO/Pt, respectively. The THz radiation is detected from the Pt-side.

In order to further quantify the spin-current generation and dissipation through very thin NiO and CoO oxide layers, we have performed THz time-domain spectroscopy. A sketch of the THz-TDS setup is shown in Figure 4a. The trilayers that are used as sources of THz radiation are excited by the strong pump beam at 800 n m . The probe beam was used to excite a photoconductive antenna (PCA) with a dipole length of 20 μ m acting as a THz detector. The spintronic emitter is magnetized by an external magnetic field with a maximum available value of 20 m T that was able to saturate the Co/CoO/Pt sample at room temperature but was not able to fully saturate the NiO sample. The pump beam was focused on the trilayer from the substrate side. In such geometry, the laser pump pulse travels through the substrate (the same for both samples) and the emission is collected from the Pt side. Since the substrates, total thicknesses and the alignment of the THz optics and detector remain the same, the measurements are relative comparable.

The emitted THz pulses for the two trilayers are shown in Figure 4c,d. The measurements were performed at room temperature under dry-air conditions. The recorded voltage is proportional to the momentary electric field amplitude of the THz wave. The bipolar pulse is the THz signal that corresponds to the spin and charge carrier dynamics. For comparison, we also show the signal strength of a reference Co (10 n m )/Pt (3 n m ) bilayer that was grown on ${\mathrm{Al}}_{2}O_{3}$ substrate. The bilayer exhibits the strongest signal, as is expected for the bilayer structure [2]. Surprisingly, the trilayers emit THz radiation. The presence of a very thin oxide barrier of 1.4 n m in thickness obviously only partially prevents the flow of a spin current. The NiO layer seems to be more susceptible to spin-current transmission, the bipolar signal is significantly above the background level. On the other hand, the Co/CoO/Pt trilayer exhibits a signal above the noise level but smaller than its NiO counterpart.

## 4. Discussion

At room temperature, the Co/CoO/Pt exhibits almost square in-plane hysteresis loops with saturation field of a few mTa nd almost 100% remanence values. The behaviour changes dramatically as we lower the temperature: hysteretic behaviour is introduced and there is a large exchange bias value below 150 K . The latter also drives the large saturation fields that can reach the value of 2 T at 4 K . The Ni/NiO/Pt exhibits slightly different magnetization reversal with an S-shape curve indicative of domain-wall nucleation when it approaches the saturation. This behaviour can be interpreted by considering the presence of a second-order uniaxial anisotropy term K_u2_ [44,57], since we do not observe 100% remanent magnetization in the hysteresis loop. Temperature-dependent measurements show that the magnetization reversal of Ni-based trilayer develops an hysteretic behaviour and small exchange bias values below 150 K .

Based on the exchange bias values, we can conclude that both the NiO and CoO layer at room temperature are not antiferromagnetically coupled with the ferromagnetic layers. However, Ni and Co layers can ferromagnetically couple the NiO and CoO layers by magnetizing them magnetostatically or through the existence of interfacial exchange coupling at the FM/CoO and NiO interfaces.

In order to quantify the spin dynamics for both trilayers, we have performed FMR measurements. We calculated the Gilbert damping parameter and we found that for the Co/CoO/Pt sample, $\alpha =1.93\times 10^{-2}$ and for the Ni/NiO/Pt sample, $\alpha =6.78\times 10^{-2}$ . Among other factors that can influence the damping values which are related to the magnetic properties of Co and Ni layers, the larger damping of the CoO and NiO samples compared to single Co and Ni layers [55,56] can also be related to better spin-current transparency through the FM/AFM and AFM/Pt interfaces. In particular, because the spin current leaving the magnetic layer carries away angular momentum from the magnetization precession to the neighbouring NM layer, it represents an additional loss channel for the magnetic system and consequently causes an additional increase in the measured Gilbert damping parameter [58]. Furthermore, the Ni/NiO/Pt seems to dissipate a larger amount of spin current, hinting at a better spin transparency for the interface. THz time-domain spectroscopy offers us a unique tool to directly probe the spin-current transport and the strength of the inverse spin Hall effect by recording the results of these processes via the THz emission.

The detection of a THz signal from the Ni/NiO/Pt shows that spin current can be transmitted through the NiO oxide layer and it can enter the Pt layer, which acts as a detector of the spin current by emitting THz radiation. The THz signal is in line with the enhanced damping that is observed of the Ni-based sample. Furthermore, the measured THz signal that is proportional to the magnetization of the sample [2] is, in reality, even larger, since the 20 m T maximum available applied external magnetic field in the THz setup is not able to fully saturate the NiO sample at room temperature; see Figure 1b. Co/CoO/Pt also exhibits a THz signal above the noise level but smaller than the NiO sample.

The oscillations of the magnetization in the GHz region of the Co and Ni ferromagnetic layers generate a flux of spin angular momentum which can be absorbed in the NiO and CoO layers. Different models have been proposed to explain how a GHzc oherent spin current can propagate coherently across an AFM insulator which inherently has THzm agnon excitations. For example, the mechanism of conduction of spin-current transfer through an AFM dielectric was attributed to the properties of an easy-axis AFM [59], or to the case in which the polarization of spin flux is parallel to the equilibrium orientation of the Néel vector of the antiferromagnetic layers [30,31]. Further, the spin-current transmission was driven by evanescent spin-wave excitations that have frequencies that are much lower than the frequency of the AFM resonance [60]; alternatively, THz thermal magnons in the AFM were responsible for the conduction [61,62].

Our CoO and NiO layers are not rigid AFM at room temperature, at which the FMR and THz measurements are taking place. In this case, the FM layer can not couple directly with the Néel vector of the AFM layer. However, the oscillations of the magnetization in the Co and Ni layers create a flux of spin angular momentum which can be absorbed into the NiO and CoO layers by creating a non-equilibrium distribution of magnons, as has been described in [30]. Damping and THz data indicate that the spin pumping from the FM layer results in the creation of non-equilibrium distribution of magnons in NiO and CoO. These magnons can absorb additional energy, and so they can contribute to the measured additional damping of the magnetic layer. The latter enables the non-resonant pumping of the spin current in the Pt layer which subsequently give rise to THz emission via the ISHE.

To further quantify the behaviour of the AFM layers when the samples are in the strong coupling regime one, has to perform low-temperature THz measurements.

## 5. Conclusions

TDS-THz measurements have revealed that thin antiferromagnetic oxide layers like NiO and CoO allow for spin-current transport at Ni/NiO-NiO/Pt and Co/CoO-CoO/Pt interfaces; however, they have different efficiencies. We attribute this to the non-resonant excitation of magnons in the AFM layers at room temperature, which is induced by spin pumping from the FM layers. NiO has a large distribution of magnons, which are able to non-resonantly transfer angular momentum to the Pt layer and subsequently to emit THz radiation. CoO can also contribute to the spin-current transparency at room temperature, but with lower efficiency. Spin-current transmission through thin antiferromagnetic layers is a highly importance topic for the future direction of antiferromagnetic spintronics.

## Data Availability

The data presented in this study are available on request from the corresponding author. The data are not publicly available due to patenting potential.

## Abbreviations

The following abbreviations are used in this manuscript:

FM	Ferromagnetic
NM	Non-magnetic
STEs	Spintronic THz emitters
THz-TDS	Terahertz time-domain spectroscopy
EB	Exchange Bias
AFM	Antiferromagnetic
